# Cellulose Nanofibers from Olive Tree Pruning as Food Packaging Additive of a Biodegradable Film

**DOI:** 10.3390/foods10071584

**Published:** 2021-07-07

**Authors:** Mónica Sánchez-Gutiérrez, Isabel Bascón-Villegas, Eduardo Espinosa, Elena Carrasco, Fernando Pérez-Rodríguez, Alejandro Rodríguez

**Affiliations:** 1 Food Science and Technology Department, Darwin Building, Universidad de Córdoba, 14014 Córdoba, Spain; q12bavii@uco.es (I.B.-V.); bt2cajie@uco.es (E.C.); b42perof@uco.es (F.P.-R.); 2 BioPrEn Group, Chemical Engineering Department, Marie-Curie Building, Universidad de Córdoba, 14014 Córdoba, Spain; a02esvie@uco.es (E.E.); a.rodriguez@uco.es (A.R.)

**Keywords:** olive tree by-products, technological properties, circular economy, valorization, bio-nanocomposite, sustainability

## Abstract

A biodegradable packaging film containing cellulose nanofibers from olive tree pruning, a by-product of olives production, was obtained using a solvent casting method. Nanocellulose was added to polyvinyl alcohol (PVA) to enhance the technological properties of the composite film as food packaging material. Nanocellulose was obtained from unbleached and bleached pulp through a mechanical and TEMPO pretreatment. Crystalline and chemical structure, surface microstructure, UV and gas barrier, optical, mechanical and antioxidant properties, as well as thermal stability were evaluated. Regarding optical properties, the UV barrier was increased from 6% for the pure PVA film to 50% and 24% for unbleached and bleached nanocellulose, respectively. The antioxidant capacity increased significantly in unbleached mechanical nanocellulose-films (5.3%) compared to pure PVA film (1.7%). In terms of mechanical properties, the tensile strength of the 5% unbleached mechanical nanocellulose films was significantly improved compared to the pure PVA film. Similarly, the 5% nanocellulose films had increased the thermal stability and improved barrier properties, reducing water vapor permeability by 38–59% and presenting an oxygen barrier comparable to aluminum layer and plastic films. Our results support the use of the developed films as a green alternative material for food packaging.

## 1. Introduction

Food packaging plays an essential role in the quality and safety of foods throughout their shelf life, protecting them from physical, chemical and biological hazards [1]. In recent decades, due to changes in consumer lifestyle, the demand for safe, high-quality, fresh, minimally processed and ready-to-eat foods, which are mainly packaged in single-use plastic packaging, has strongly increased, resulting in a negative environmental impact [2,3].

Plastics are the most widely used material in food packaging, with more than 30% of worldwide production destined for this application. Global demand and the responsive production of plastic materials for food packaging has increased considerably over the last six decades and is expected to continue for the next 20 years [4,5]. Petroleum-based polymeric plastic materials such as polyvinylchloride (PVC), polyethylene terephthalate (PET), polypropylene (PP), polyethylene (PE), polyamide (PA), polystyrene (PS) and ethylene vinyl alcohol (EVOH), are widely used in food packaging due to their good mechanical and barrier properties, low cost and high availability [6]. However, the high negative impact on the environment caused by low degradability of such materials has led to increased global concern; indeed, over ten megatons (Mt) of plastic waste ends up in the oceans [1,7].

Innovations in the food packaging industry have focused on the development of new biodegradable packaging materials, for which demand has increased considerably in past years. The market for biopolymer-based packaging materials is expected to grow up to 16.8 billion dollars by 2022. Nevertheless, their use is limited within the food industry because of their poor mechanical and barrier properties [4].

Polyvinyl alcohol (PVA) is a semicrystalline, nontoxic, water-soluble, biodegradable and therefore environmentally friendly synthetic polymer used in food packaging [8,9]. However, due to its hydrophilic nature, it is characterized by its low resistance to humid environments, leading to a decrease in its oxygen barrier and mechanical properties [10]. Therefore, the use of PVA films for food packaging would only be suitable in nonmoisture environments [11]. To improve its mechanical and barrier properties, PVA could be combined with other substances used as additives, such as biomass-derived biopolymers like cellulose nanofibers (CNFs) [12].

Nanocellulose, isolated from cellulose, constitutes one of the most abundant, low-cost and biodegradable natural biopolymers with several industrial applications, including its use as reinforcement material in polymeric matrices, previously documented by several authors who incorporated nanocellulose from different plant by-products, not including olive tree pruning [11,13,14,15,16]. The presence of hydroxyl groups in cellulose nanofibers, and even some aromatic hydroxyls of lignin in the case of lignocellulose nanofibers, suggests a good interface between the polymeric matrix and reinforcement. Nanocellulose is becoming increasingly important in the food packaging industry, mainly due to its sustainable and environmentally friendly production from agricultural waste [17,18]. Globally, agricultural activity yields a large amount of highly available and low-cost by-products that can be used to obtain added-value products. However, most of these by-products are currently not valorised and end up in landfills, entailing environmental damage and economic costs. In this sense, as an alternative to the linear economy (take-make-use-dispose), the circular economy (grow-make-use-restore) is proposed, in which the valorisation of by-products plays a key role and constitutes a major challenge [19,20,21].

Spain represents 31% of total worldwide olive production, dedicating 2.8 M ha to olive tree cultivation, equivalent to 26% of the world cultivation area [22]. This production generates more than 7.5 million tons of lignocellulosic waste per year, including olive leaf and olive tree pruning, the latter generated during olive grove maintenance work. Traditionally, this waste has no industrial applications and is usually burned or used as soil fertilizer. A better utilization of these products can be made, adding value, and reintroducing them into the economic cycle [23,24].

In this research, (ligno)cellulose nanofibers ((L)CNFs) from olive tree pruning were used to investigate the influence of residual lignin and pretreatment on the reinforcing effect of cellulose nanofibers on the PVA matrix with the aim of developing a film with potential for food applications. The physico-chemical properties of the PVA-(L)CNF bionanocomposite films were studied, and the effects of (L)CNF on the optical, antioxidant, barrier, mechanical and thermal properties of the films were investigated.

## 2. Materials and Methods

### 2.1. Materials

Olive tree pruning material was obtained after the annual pruning of an olive tree grove in the province of Córdoba (Spain), following olive harvest.

Polyvinyl alcohol (PVA) (M.W.: 146,000–186,000; and degree of hydrolysis + 99%), 2,2,6,6-piperidin-1-oxyle TEMPO and ABTS diammonium salt (2,2-azino-bis (3-ethylbenzothiazoline-6-sulphonic acid)) were obtained from Sigma-Aldrich (Madrid, Spain). Sodium hydroxide was acquired from Panreac (Castellar del Vallès, Barcelona, Spain) and sodium hypochlorite from Honeywell (Charlotte, NC, USA).

### 2.2. Pulping Process and Isolation of Nanocellulose

Olive tree pruning material was subjected to a pulping process using 16% (over dried matter) NaOH as a reagent, at 170 °C for 60 min and then the pulp was subjected to a bleaching process as described by Sánchez-Gutiérrez et al. [23].

Unbleached (U) and bleached pulp (B) were used to obtain CNFs using two different pretreatments: mechanical (M) and TEMPO-mediated oxidation (T), both followed by a high-pressure homogenisation treatment [15].

### 2.3. Preparation of PVA/(Ligno)nanocellulose Films

The PVA-(L)CNF films were prepared by the solvent casting method. The PVA solution (3 wt%) was dissolved in distilled water at 90 °C for 4 h by mechanical stirring. Three concentrations of the CNF suspension (2.5, 5 and 7.5% (*w*/*w*)) were added to the PVA solution, which were mixed under continuous stirring at room temperature for 4 h. The bionanocomposite films were prepared by casting the suspensions into 14 cm diameter Petri plates and drying at room temperature until a dry weight of 0.35 g per film was reached. Finally, the dried films were peeled from the casting surface and preconditioned (23 °C with 50% RH for 24 h) prior to characterization.

### 2.4. Characterization

#### 2.4.1. Optical Properties

The light transmittance of the film in the UV-VIS regions (200–800 nm) was determined with a Perkin Elmer UV/VIS Lambda 25 spectrophotometer (Waltham, MA, USA), using the following equations to assess the UV barrier (Equation (1)) and transparency properties (Equation (2)) of the films by means of the transmittance values of the films at wavelengths of 280 nm and 660 nm, which are consistent with the UV-VIS absorption spectra [25].

*UV-barrier* = 100 − (%*T*_280_/%*T*_660_) × 100 (1)

where %*T*_280_ and %*T*_660_ are the transmittance percentage at 280 nm and 660 nm, respectively.

*Transparency* = log %*T*_660_/*x* (2)

where %*T*_660_ is the transmittance percentage at 660 nm and *x* is the thickness of the film (mm).

#### 2.4.2. Fourier Transform Infrared (FTIR) Spectroscopy

The spectra of the films were obtained using a FTIR-ATR Perkin-Elmer Spectrum Two (Waltham, MA, USA) with a resolution of 4 cm^−1^ in the range of 500 to 4000 cm^−1^. FTIR spectra were compared to evaluate the effects of nanocellulose incorporated in the PVA films based on the intensity and shift of vibrational bands. A total of 40 scans were collected for each sample.

#### 2.4.3. Thermogravimetric Analysis (TGA)

The thermal stability of the pure PVA and PVA-(L)CNF films was assessed by thermogravimetric analysis (TGA) using a Toledo Thermogravimetric analyzer (TGA/DSC 1) (Mettler Toledo, L’Hospitalet de Llobregat, Barcelona, Spain). The measurements were performed by heating the film samples (10.0 ± 1.0 mg) from room temperature to 600 °C at a heating rate of 10 °C/min under a nitrogen atmosphere with a nitrogen gas flow of 50 mL/min. The temperature at which the degradation rate reached its maximum (T_max_), was evaluated by analyzing the TGA equivalent derivate (DTG).

#### 2.4.4. Scanning Electron Microscopy (SEM)

The surface and cross-sectional morphology of the pure PVA and PVA-(L)CNF films were analyzed using a JEOL JSM-7800F Scanning Electron Microscopy (SEM). All film samples were coated using conductive gold sputter at 10 kV.

#### 2.4.5. X-ray Diffraction (XRD) Analysis

The X-ray diffraction patterns of the composites were obtained using a Bruker D8 Discover with a monochromatic source CuKα1 between angular range of 5–50° at a 1.56°/min scan speed in order to evaluate the changes in crystallinity after addition of (L)CNFs to PVA films.

#### 2.4.6. Antioxidant Activity

The ABTS scavenging activity assay of samples was determined as described by Espinosa et al. [15]. A radical solution was prepared (7 mM ABTS and 2.45 mM persulphate of potassium) and left in the dark overnight for 12–16 h before use. The radical solution was diluted with ethanol to an absorbance of 0.70 ± 0.02 at 734 nm. An aliquot of this solution (4 mL) was mixed with 1 cm^2^ of the films and, after 6 min, the absorbance of the samples was measured with a spectrophotometer at 734 nm. The percentage of reduction of ABTS_734_ (antioxidant power (AOP)) was calculated with the Equation (3).

*AOP* = (*A_ABT_*_S6’_ − *A_ABTSfilm6_*_´_/*A_ABTS0_*_´_) × 100 (3)

where *A_ABTS6_*_´_ is the absorbance at 734 nm of the ABTS radical solution after 6 min, *A_ABTSfilm6_*_´_ is the absorbance at 734 nm of the sample after 6 min and *A_ABTS_*_0´_ is the initial absorbance at 734 nm of the ABTS radical solution (0 min). ABTS scavenging activity was expressed as %AOP per gram of the film. All assays were performed in triplicate.

#### 2.4.7. Mechanical Properties

An LF Plus Lloyd Instrument (AMETEK Measurement & Calibration Technologies Division, Largo, FL, USA) provided with a 1 kN load cell was used to calculate the tensile strength, elongation at break, traction and Young’s modulus of the pure PVA and PVA-(L)CNF films according to the ASTM D638 standard test method [26]. Film samples were cut into 100 × 15 mm strips and then preconditioned at 25 °C for 48 h and 50% RH before the test. The initial distance was set at 65 mm and the stretching rate at 10 mm/min. The thickness of the films at five random positions was measured using a Digital Micrometre IP65 0-1”, (Mitutoyo, Neuss, Germany) with a sensitivity of 0.001 mm. At least five samples at each CNF concentration were tested.

#### 2.4.8. Barrier Properties: Water Vapor Permeability and Oxygen Transmission Rate (WVP and OTR)

The Water Vapor Permeability (WVP) of the films was measured according to the ASTM E96/E96M-10 standard method [27]. Square film samples of 4 cm^2^ were attached with aluminum adhesive tape to containers, whose lids were punched with a circular cork borer of 10 mm diameter and containing CaCl_2_ as desiccant material. Then, containers were placed in a controlled chamber at 25 °C and 50 ± 2% RH. In order to evaluate mass gain, the weight of the containers was measured at different time intervals for 24 h and used to calculate the water vapor transmission rate (WVTR) using Equation (4):

*WVTR =* (*G/t × A*) (4)

where *G* is the weight change (g) at time *t* (h) and *A* is the test area (m^2^)

The WVTR was used to calculate de WVP coefficient according to Equation (5).

*WVP = WVTR × Th/P*
× (*RH_out_ − RH_in_*) (5)

where *Th* is the thickness of the film (m), *P* is the saturation vapor pressure at the test temperature (Pa), *RH_out_* is the relative humidity outside the container (50%) and *RH_in_* is the relative humidity inside the container where the CaCl_2_ is placed (0%).

The Oxygen Transmission Rate (OTR) of the films was measured using a Mocon OX-TRAN^®^ 2/22 OTR analyser (Mocon, MN, USA) at 23 °C and 50% RH. The test was run according to the ASTM D3985-17 standard method by measuring the steady-state transmission rate of oxygen gas through the bionanocomposite using an oxygen-sensitive coulometric sensor operating at constant performance to monitor the amount of oxygen transmitted. For the measurement, oxygen gas was applied to one side of the barrier material and nitrogen gas was applied to the other side. For this purpose, the test film was placed between the two sides of the test cell. As the oxygen gas penetrated the film, the nitrogen carrier gas was transported into the coulometric detector [28].

### 2.5. Statistical Analysis

In order to evaluate the influence of the bleaching process, pretreatment and CNF concentration in the film’s formulation, one-way analysis of variance (ANOVA) and Tukey’s post hoc test were carried out using the IBM^®^ SPSS^®^ Statistics software Version 25 (IBM Corporation, New York, NY, United States), with a significance level of *p* < 0.05. All data are reported as mean ± standard deviation.

## 3. Results and Discussion

### 3.1. Structure Characterization

The micrographs obtained by SEM showed the microstructure and dispersion of the nanocellulose in the PVA film.

**Figure 1:**
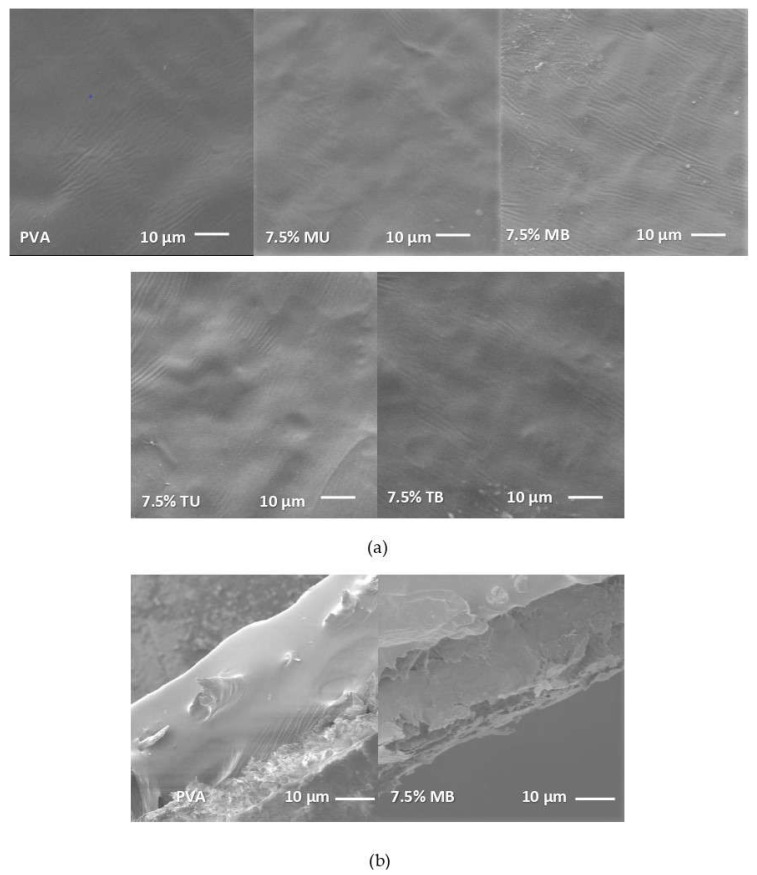
SEM micrographs of pure PVA and PVA-(L)CNF films. (**a**) surface of pure PVA, PVA containing 7.5% of mechanical unbleached nanocellulose (7.5% MU), PVA containing 7.5% of mechanical bleached nanocellulose (7.5% MB), PVA containing 7.5% of TEMPO unbleached nanocellulose (7.5% TU), PVA containing 7.5% of TEMPO bleached nanocellulose (7.5% TB); (**b**) cross-section of pure PVA and PVA containing 7.5% of mechanical bleached nanocellulose (7.5% MB).

As can be seen in Figure 1a, the scanning electron micrographs of the PVA film’s surface containing 7.5% of nanocellulose showed a distribution pattern rougher than that of the pure PVA film. It was observed that the higher the nanocellulose concentration, the higher the surface roughness of the PVA-(L)CNF films, due to the increased agglomeration of the nanofibers. In relation to the lignin content, it was observed that films with unbleached nanocellulose (MU and TU), due to their lignin content, showed greater roughness than films obtained with bleached nanocelluloses (MB and TB) [29]. According to the type of pretreatment, i.e., mechanical or TEMPO, differences were observed depending on the presence or not of lignin in the nanocellulose fibers. While the mechanical treatment resulted in films with smoother surfaces when lignin was present (MU vs. TU), the TEMPO pretreatment yielded smoother film surfaces in the case of bleached fibers (TB vs. MB). In both cases, this surface texture was probably the result of the lower diameter of the nanofibers, since in mechanical pretreatment the presence of lignin resulted in a higher fibrillation yield, whereas in TEMPO pretreatment the absence of lignin led to more effective oxidation [15,23].

The distribution and integration of the nanocellulose in the PVA matrix were evaluated by means of a cross section analysis. As can be observed in Figure 1b, the nanocellulose was homogeneously distributed with no (L)CNF precipitation zones observed during the dissolving-casting process, showing a well dispersed distribution pattern with no layer separation.

### 3.2. Chemical Structure

**Figure 2:**
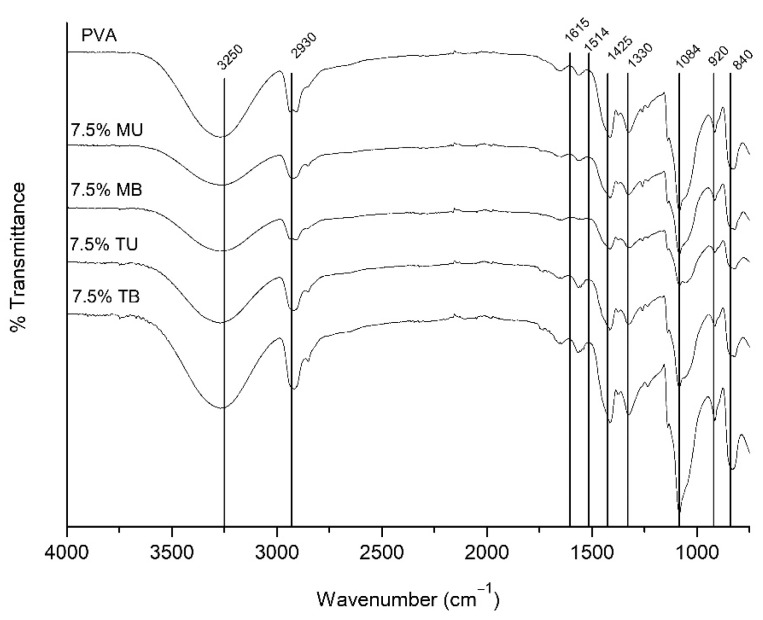
ATR-FTIR spectra of pure PVA and PVA-(L)CNF films (PVA containing 7.5% of mechanical unbleached nanocellulose (7.5% MU), PVA containing 7.5% of mechanical bleached nanocellulose (7.5% MB), PVA containing 7.5% of TEMPO unbleached nanocellulose (7.5% TU) and PVA containing 7.5% of TEMPO bleached nanocellulose (7.5% TB)).

Figure 2 shows the FTIR spectra of pure PVA and 7.5% PVA-(L)CNF films, revealing that the spectral pattern of pure PVA was very similar to the spectra of the films reinforced with 7.5% CNF. This fact would indicate that the addition of CNF had no influence on the molecular structure of the PVA, with the chemical structure remaining stable, with no changes.

All spectra showed peaks at 3250 cm^−1^ attributed to the typical O-H stretching vibration from intermolecular and intramolecular hydrogen bonds. The peak corresponding to the C-H stretching vibrations of methyl or methylene groups was observed at around 2930 cm^−1^. The vibration peak detected at 1425 cm^−1^ has been related to the bending mode of CH_2_ bonds. The peaks at 1330 and 1084 cm^−1^ were associated with C-O stretching, the vibration around 920 cm^−1^ characterized the CH_2_ groups, while the absorption band at 840 cm^−1^ is related to the stretching vibration of the C-C groups [13,30].

The slight differences found between the spectra of pure PVA and the PVA-(L)CNF films were due to the different chemical composition of the nanocelluloses and were observed in the 1615 and 1514 cm^−1^ peaks associated with C=O stretching vibration in carboxyl groups, and the C=C of the lignin aromatic rings, respectively. The peak at 1615 cm^−1^ was more intense in the films reinforced by TEMPO CNF (TU and TB) as a consequence of carboxyl groups enhancement by the action of TEMPO oxidation, while the peak at 1514 cm^−1^ was less intense in the films reinforced with TU, TB and MB CNF, owing to the oxidation of lignin in TEMPO pretreatment and removal in the bleaching step [15,31].

### 3.3. Crystalline Structure

**Figure 3:**
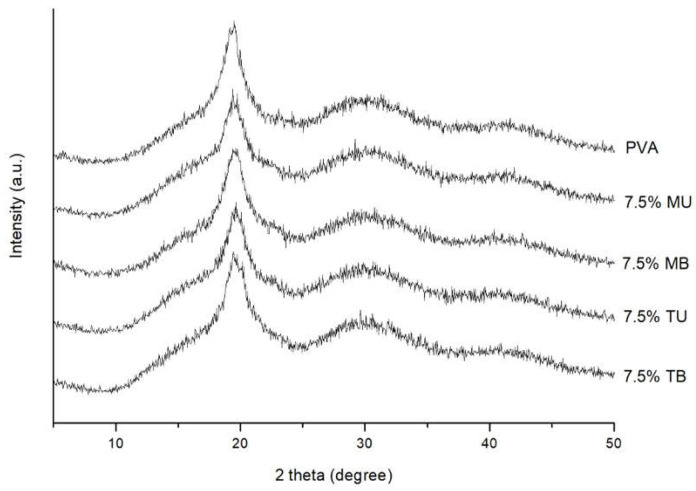
X-ray diffractograms of pure PVA and PVA-(L)CNF films.

To evaluate the crystalline and amorphous regions of pure PVA and 7.5% PVA-(L)CNF films, X-ray diffraction patterns were studied (Figure 3). The pure PVA film showed the typical strong semicrystalline structure of PVA, showing an X-ray diffractogram with a typically strong crystalline signal at 19.7° due to hydrogen bonds between the hydroxyl groups of the PVA chains, and a broad signal at 19.5° corresponding to the amorphous section [14].

The 7.5% PVA-(L)CNF films showed signals with higher intensity between 19.4° and 19.7°, similar to that of pure PVA, indicating that the incorporation of (L)CNFs did not modify the semicrystalline structure of PVA. It was also confirmed that the addition of CNF did not show significant changes in the position of the peaks. In olive tree pruning (L)CNFs, the known diffraction signal at 16.1° and 22.5°, corresponding to the 110 and 200 reflection planes of cellulose I structure [16,23], practically disappeared in the composite films of this study.

### 3.4. Optical Properties

UV light in the range of 200 to 280 nm is one cause of lipid oxidation of foodstuffs, which is a problem for the food industry. Therefore, the development of films with barrier properties against UV light would help to prevent, or at least slow down, UV light lipid oxidation [32].

**Figure 4:**
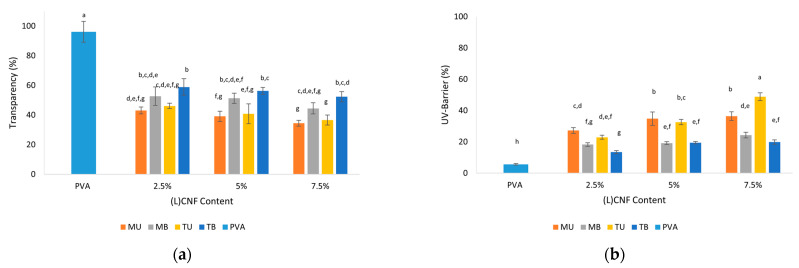
Optical properties of pure PVA and PVA-(L)CNF films formulated with different concentrations. (**a**) Transparency (%); (**b**) UV-barrier (%). Different letters above the bars represent groups significantly different at *p* < 0.05.

In this work, the optical properties of pure PVA and PVA-(L)CNF films were determined by measuring the transmittance of light in the UV and visible ranges in order to analyze the transparency and UV barrier of the films. As can be seen in Figure 4a,b, the effect of CNF type, CNF concentration and their interaction on the UV barrier was statistically significant (*p* < 0.05), as was transparency, except for their interaction (*p* > 0.05).

To a large extent, the optical transmittance of the films depends on the dispersion of the nanocellulose in the PVA matrix. As shown in Figure 4a, the films reinforced with CNF were less transparent than the pure PVA film, with a minimum transparency value of 34.5% in 7.5% MU PVA-CNF film versus 96.1% for pure PVA (*p* < 0.05). Regarding the type of CNF, it can be observed that the addition of unbleached CNF obtained by TEMPO pretreatment (TU) in the PVA matrix led to films with lower transparency than the bleached ones (TB) (*p* < 0.05). This reduction in transparency was likely due to the slight brown color of the unbleached TEMPO PVA-(L)CNF films, which contains opaque lignin [29]. However, those obtained by mechanical pretreatment (MU and MB) did not show significantly different transparency values (*p* > 0.05). In relation to the concentration of CNF, the transparency values were higher at lower concentration (2.5%), but without significant differences at the remaining concentration (5% and 7.5%) (*p* > 0.05).

In contrast, as can be observed in Figure 4b, the pure PVA film exhibited a lower UV-barrier (5.7%) than the PVA-(L)CNF films (*p* < 0.05). Overall, the incorporation of higher concentrations of CNF in the PVA matrix increased the capacity of UV absorption, with the 7.5% TU PVA-CNF film exhibiting a maximum value of 48.8% (*p* < 0.05). The UV- barrier values of the unbleached PVA-CNF films (MU and TU) were higher than the bleached ones (MB and TB), showing their greater UV absorption capacity (*p* < 0.05). The enhanced UV light blocking of the unbleached PVA-CNF films can be explained by the strong UV-absorption behavior of the lignin, mainly by the chromophore groups present in the lignin [33].

### 3.5. Antioxidant Activity

**Figure 5:**
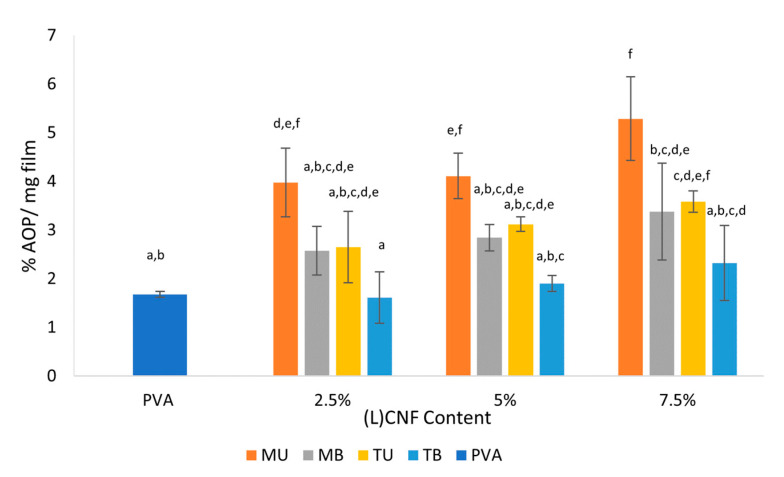
Antioxidant power (AOP) of pure PVA and PVA-(L)CNF films formulated with different concentrations. Different letters above the bars represent groups significantly different at *p* < 0.05.

The antioxidant activity of the films is an important functional feature as it can prevent the oxidative spoilage of foods through packaging [34]. Figure 5 shows the antioxidant activity of pure PVA and PVA-(L)CNF films measured by the ABTS assay and expressed as percentage of antioxidant power (AOP). The effect of CNF type and concentration was statistically significant (*p* < 0.05), whereas the interaction of both was not (*p* > 0.05). The AOP values of the unbleached CNF-reinforced films obtained by mechanical pretreatment (2.5, 5 and 7.5% MU) and unbleached 7.5% CNF TEMPO (7.5% TU) were significantly higher (*p* < 0.05) compared to pure PVA film, the latter showing the lowest value (1.7% AOP/mg film), and the 7.5% MU PVA-CNF film the highest value (5.29% AOP/mg film). These results suggest that the higher residual lignin content present in unbleached mechanical nanocellulose results in its higher antioxidative activity, mainly attributed to its phenolic hydroxyl groups, which would reduce the ABTS radical through electron transfer. In addition, the mechanical pretreatment would not affect the oxidation status of the composite material in comparison with the TEMPO pretreatment, favoring the antioxidant capacity of the former [35]. Although TEMPO pretreatment is carried out to perform a selective oxidation of the OH groups of cellulose, the sodium hypochlorite used as an initiating agent for catalytic oxidation can produce secondary and undesired oxidation of the lignin present in the fiber, which would cause a solubilization of the lignin, and therefore a bleaching of the fiber, reducing the lignin content in the fiber and thus the antioxidant capacity of the material. This would explain why the TU PVA-CNF films, despite having residual lignin content, exhibited a similar range to the MB PVA-CNF films in terms of antioxidant activity. Espinosa et al. [15] described that PVA films reinforced with wheat straw (ligno)nanocellulose showed up to twice the AOP compared to pure PVA film, values similar to those described in this work, while other authors reported how the incorporation of TEMPO-bleached CNF from eucalyptus pulp into a mucilage-based composite could significantly improve the antioxidant characteristics of the film almost three-fold compared to control [36].

### 3.6. Mechanical Properties

**Figure 6:**
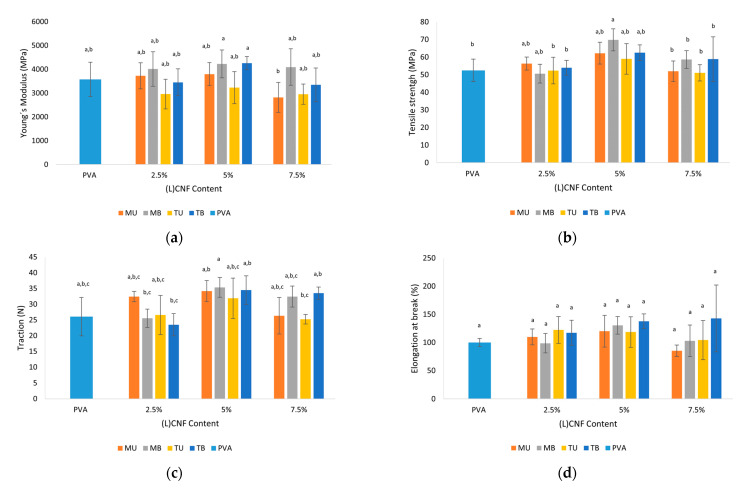
Mechanical properties of pure PVA and PVA-(L)CNF films. (**a**) Young’s modulus; (**b**) tensile strength; (**c**) traction and (**d**) elongation at break. Different letters above the bars represent groups significantly different at *p* < 0.05.

Since the use of PVA for food packaging applications is limited due to its poor mechanical properties, the effect of incorporating different (L)CNFs as reinforcement of PVA films was studied. As illustrated in Figure 6, in general terms, the mechanical properties (Young´s modulus, tensile strength, traction and elongation) were improved by the incorporation of the different (L)CNFs in the PVA matrix, but not significantly. A significant enhancement was observed in the tensile strength of the 5% MB PVA-CNF film, which can be explained by the compact structure of PVA, the stiffness of the nanocellulose chain, the homogeneous distribution of the CNF in the PVA matrix and the strong interaction and hydrogen bonding between OH groups of the nanocellulose and the PVA [14].

The type of nanocellulose significantly affected the Young’s modulus and elongation at break values (*p* < 0.05), as opposed to tensile strength and traction (*p* > 0.05). The incorporation of different concentrations of CNF was significant except for elongation at break, while the interaction of nanocellulose type and percentage was not significant (*p* > 0.05), except for tensile strength.

Regarding Young’s modulus (Figure 6a), the mean value of pure PVA film was 3578 MPa. Higher mean values were observed in films with 5% CNF TB and MB (4263 and 4229 MPa, respectively), although the differences with PVA were not significant (*p* > 0.05). Figure 6b shows the tensile strength value of the films assayed, with the 5% MB PVA-CNF film as the only bionanocomposite exhibiting a significant increase over the pure PVA film (69.8 MPa versus 52.5 MPa). With reference to traction and elongation at break, none of the films showed significantly improved values, although slightly higher mean values were observed in the 5% MB PVA-CNF film for traction (35.4 N), and in the 7.5% TB PVA-CNF for elongation at break value (143%) (Figure 6c,d).

In general, the slightly higher values of mechanical properties in MU and MB PVA-CNF films could be attributed to the high aspect ratio of these (L)CNFs. In terms of concentration, a decreasing trend in the mechanical properties of the films was observed when the (L)CNF loading was higher than 5%. This behaviour at concentrations over 5% can be explained by the higher agglomeration rate of nanocellulose in PVA-(L)CNF films acting as weak points of the films due to the breakdown of the interaction between CNF and the PVA matrix [37].

### 3.7. Barrier Properties

Determination of barrier properties is crucial in the development of food packaging. The type of specific barrier needed depends on the type of food commodity. For example, in most foods, an effective barrier against moisture and oxygen will increase the quality and shelf-life of a food product [38].

The water vapor and oxygen barrier properties of pure PVA and 5% PVA-(L)CNF films were evaluated. As can be seen in Table 1, PVA-(L)CNF films showed a lower water vapor permeability (WVP) (*p* < 0.05) compared to pure PVA films. The type of nanocellulose incorporated as reinforcement influenced the reduction of WVP (*p* < 0.05). In relation to the bleaching treatment, it was observed that the WVP in the unbleached PVA-CNF films (MU and TU) was higher than in the bleached ones (MB and TB), while in terms of the influence of the pretreatment, the nanocellulose reinforced films obtained by TEMPO (TU and TB) showed the lowest WVP values compared to the mechanical nanocellulose reinforced films (MU and MB). A drastic reduction of WVP was evident in the 5% TB PVA-CNF film, which showed the lowest WVP value (2.82 × 10^−7^ g/s·m·Pa) compared to the pure PVA film (6.97 × 10^−7^ g/s·m·Pa) (*p* < 0.05). The reduction of WVP in PVA-(L)CNF films could be attributed to the network formed through hydrogen bonds between PVA and (L)CNFs reducing the free space in the PVA matrix and sealing the gaps to the passage of water vapor across the film [39]. However, the presence of lignin could hinder these bonds, creating more hydrophobic pores that would facilitate water vapor passage through the film, which would explain the poor aptitude as a barrier to water vapor of LCNF (MU and TU) compared to CNF (MB and TB) [40].

As commented previously, the oxygen permeability of food packaging is highly important for food preservation, extending its shelf-life and preserving its quality. The oxygen transmission rate (OTR) values showed that PVA films reinforced with 5% CNF were impermeable to oxygen, i.e., with a high oxygen barrier. While the pure PVA film had a value of 3.75 cc/m^2^·24 h, the films reinforced with MU, TU and TB CNF showed a high oxygen barrier comparable to an aluminum layer, whereas the film reinforced with MB CNF presented a good oxygen barrier, comparable to plastic films such as PET AlO_X_ and PET SiO_X_. The lower oxygen permeability of PVA-(L)CNF films may be due to the inherent flexibility of CNF, which are able to form a denser film, sealing most of the spaces between fibrils where oxygen molecules would penetrate more slowly and with more difficulty [40]. In addition, the presence of lignin would contribute to a higher compaction of the film structure, making it less porous and less permeable to oxygen, which would account for the higher oxygen barrier of the unbleached PVA-CNF films (MU and TU) compared to the bleached ones (MB and TB) [15]. It is important to highlight that (L)CNF-reinforced films obtained by mechanical pretreatment (MU and MB), because are more susceptible to environmental conditions such as humidity and temperature, required a longer stabilization time than (L)CNF-reinforced films obtained by TEMPO pretreatment (TU and TB). This behavior occurs in other materials such as EVOH, which are greatly affected by the ambient humidity. To overcome this limitation, this material must be sandwiched by creating a barrier layer through lamination or coextrusion, thus keeping its barrier function [41,42].

### 3.8. Thermal Stability

The evaluation of thermal properties is necessary to estimate the practical applications of PVA-(L)CNF films for use in the agrifood industry. The thermal stability of pure PVA and 5% PVA-(L)CNF films was measured by calculating the maximum degradation temperature (T_max_) through the derivative of an TGA curve (DTG). As can be seen in Table 2, PVA showed the lowest maximum degradation temperature (250.32 °C), while the film reinforced with 5% MU PVA-CNF exhibited the highest maximum degradation temperature (264.99 °C). It was observed that the absence of lignin (MB and TB) resulted in films with lower T_max_ than the MU and TU films, while the type of nanocellulose pretreatment did not influence the T_max_ value since very similar values were reported for the MU-TU and MB-TB films. The influence of lignin, present in the unbleached samples, on T_max_, may be explained by the covalent bonds with cellulose providing high thermal stability to the fibers [43].

## 4. Conclusions

PVA films reinforced with (L)CNFs from olive tree pruning waste were obtained by a solvent casting method. Two different pretreatments (mechanical and TEMPO) were used on bleached and unbleached cellulose pulps to isolate nanocellulose. The incorporation of (L)CNFs did not modify the physical and chemical structure of the PVA matrix due to the homogeneous dispersion of the (L)CNFs within the matrix. However, antioxidant activity increased significantly after the addition of unbleached mechanical CNFs due to the higher lignin content. Similarly, the UV barrier properties were enhanced significantly with the incorporation of all types of (L)CNFs, resulting in films with lower transparency but higher UV-light blocking capacity. In addition, the introduction of nanocellulose improved water vapor and oxygen barriers, decreasing their values, with a drastic fall of the OTR. The thermal stability of 5% PVA-(L)CNF films was also higher in comparison to pure PVA. The addition of (L)CNFs slightly increased the values of the mechanical parameters measured, with the addition of 5% mechanical bleached nanocellulose (MB) causing a significant improvement in terms of tensile strength. The films developed in this work could be potentially employed in the food packaging sector, providing additional technological benefits. The barrier properties against UV, water vapor and oxygen, together with the proven antioxidant activity necessary for preservation of several food commodities, including the prevention of lipids oxidation in foods, would confer on the developed bionanocomposites the optimum functionalities for food stabilization during shelf-life. Further studies should be considered to investigate the mechanisms of biodegradation as well as the possible migration of film components into food.

## Figures and Tables

**Table 1:** Barrier properties of pure PVA and 5% PVA-(L)CNF films.

Films	WVP (10^−7^.g/s·m·Pa)	OTR (cc/m^2^⋅Day)
PVA	6.97 ± 0.07 ^a^	3.75
5% MU	4.31 ± 0.31 ^b^	0.08
5% MB	4.16 ± 0.02 ^b^	0.64
5% TU	3.88 ± 0.07 ^b^	0.02
5% TB	2.82 ± 0.17 ^c^	0.06

Different superscript letters indicate values significantly different (*p* < 0.05) according to Tukey´s Multiple Range Test.

**Table 2:** T_max_ of pure PVA and 5% PVA-(L)CNF films.

Films	T_max_ (°C)	Residue Mass at 600 °C (%)
PVA	250.32	12
5% MU	264.99	8
5% MB	259.12	1
5% TU	263.39	5
5% TB	257.52	1

## Data Availability

The data presented in this study are available on request from the corresponding author.

## References

[1] Bhargava N., Sharanagat V.S., Mor R.S., Kumar K. (2020). Active and intelligent biodegradable packaging films using food and food waste-derived bioactive compounds: A review. Trends Food Sci. Technol..

[2] Soltani Firouz M., Mohi-Alden K., Omid M. (2021). A critical review on intelligent and active packaging in the food industry: Research and development. Food Res. Int..

[3] Chakori S., Aziz A.A., Smith C., Dargusch P. (2021). Untangling the underlying drivers of the use of single-use food packaging. Ecol. Econ..

[4] Pinto L., Bonifacio M.A., De Giglio E., Santovito E., Cometa S., Bevilacqua A., Baruzzi F. (2021). Biopolymer hybrid materials: Development, characterization, and food packaging applications. Food Packag. Shelf Life.

[5] Mendes A.C., Pedersen G.A. (2021). Perspectives on sustainable food packaging:– is bio-based plastics a solution?. Trends Food Sci. Technol..

[6] Asgher M., Qamar S.A., Bilal M., Iqbal H.M.N. (2020). Bio-based active food packaging materials: Sustainable alternative to conventional petrochemical-based packaging materials. Food Res. Int..

[7] Sundqvist-Andberg H., Åkerman M. (2021). Sustainability governance and contested plastic food packaging e An integrative review. J. Clean. Prod..

[8] Ma Q., Liang T., Cao L., Wang L. (2018). Intelligent poly (vinyl alcohol)-chitosan nanoparticles-mulberry extracts films capable of monitoring pH variations. Int. J. Biol. Macromol..

[9] de Carvalho S.M., Noronha C.M., da Rosa C.G., Sganzerla W.G., Bellettini I.C., Nunes M.R., Bertoldi F.C., Manique Barreto P.L. (2019). PVA antioxidant nanocomposite films functionalized with alpha-tocopherol loaded solid lipid nanoparticles. Colloids Surf. A Physicochem. Eng. Asp..

[10] Lee H., You J., Jin H.J., Kwak H.W. (2020). Chemical and physical reinforcement behavior of dialdehyde nanocellulose in PVA composite film: A comparison of nanofiber and nanocrystal. Carbohydr. Polym..

[11] Srivastava K.R., Dixit S., Pal D.B., Mishra P.K., Srivastava P., Srivastava N., Hashem A., Alqarawi A.A., Abd_Allah E.F. (2021). Effect of nanocellulose on mechanical and barrier properties of PVA–banana pseudostem fiber composite films. Environ. Technol. Innov..

[12] Yang W., Ding H., Qi G., Li C., Xu P., Zheng T., Zhu X., Kenny J.M., Puglia D., Ma P. (2021). Highly transparent PVA/nanolignin composite films with excellent UV shielding, antibacterial and antioxidant performance. React. Funct. Polym..

[13] Mandal A., Chakrabarty D. (2014). Studies on the mechanical, thermal, morphological and barrier properties of nanocomposites based on poly(vinyl alcohol) and nanocellulose from sugarcane bagasse. J. Ind. Eng. Chem..

[14] Singh S., Gaikwad K.K., Lee Y.S. (2018). Antimicrobial and antioxidant properties of polyvinyl alcohol bio composite films containing seaweed extracted cellulose nano-crystal and basil leaves extract. Int. J. Biol. Macromol..

[15] Espinosa E., Bascón-Villegas I., Rosal A., Pérez-Rodríguez F., Chinga-Carrasco G., Rodríguez A. (2019). PVA/(ligno)nanocellulose biocomposite films. Effect of residual lignin content on structural, mechanical, barrier and antioxidant properties. Int. J. Biol. Macromol..

[16] Sarwar M.S., Niazi M.B.K., Jahan Z., Ahmad T., Hussain A. (2018). Preparation and characterization of PVA/nanocellulose/Ag nanocomposite films for antimicrobial food packaging. Carbohydr. Polym..

[17] Amara C., El Mahdi A., Medimagh R., Khwaldia K. (2021). Nanocellulose-based composites for packging applications. Curr. Opin. Green Sustain. Chem..

[18] Liu Y., Ahmed S., Sameen D.E., Wang Y., Lu R., Dai J., Li S., Qin W. (2021). A review of cellulose and its derivatives in biopolymer-based for food packaging application. Trends Food Sci. Technol..

[19] Karimi A., Kazemi M., Samani S.A., Simal-Gandara J. (2021). Bioactive compounds from by-products of eggplant: Functional properties, potential applications and advances in valorization methods. Trends Food Sci. Technol..

[20] Bascón-Villegas I., Espinosa E., Sánchez R., Tarrés Q., Pérez-Rodríguez F., Rodríguez A. (2020). Horticultural plant residues as new source for lignocellulose nanofibers isolation: Application on the recycling paperboard process. Molecules.

[21] Barros M.V., Salvador R., de Francisco A.C., Piekarski C.M. (2020). Mapping of research lines on circular economy practices in agriculture: From waste to energy. Renew. Sustain. Energy Rev..

[22] FAOSTAT (2021). http://www.fao.org/faostat/en/#data/QC/visualize.

[23] Sánchez-Gutiérrez M., Espinosa E., Bascón-Villegas I., Pérez-Rodríguez F., Carrasco E., Rodríguez A. (2020). Production of cellulose nanofibers from olive tree harvest—A residue with wide applications. Agronomy.

[24] Gullón B., Gullón P., Eibes G., Cara C., De Torres A., López-Linares J.C., Ruiz E., Castro E. (2018). Valorisation of olive agro-industrial by-products as a source of bioactive compounds. Sci. Total Environ..

[25] Rincón E., Serrano L., Balu A.M., Aguilar J.J., Luque R., García A. (2019). Effect of bay leaves essential oil concentration on the properties of biodegradable carboxymethyl cellulose-based edible films. Materials.

[26] (2014). ASTM D638-14 Standard Test Method for Tensile Properties of Plastics.

[27] (2010). ASTM-E96/E96M-10 Standard Test Methods for Water Vapor Transmission of Materials.

[28] (2017). ASTM D3985—17 Standard Test Method for Oxygen Gas Transmission Rate Through Plastic Film and Sheeting Using a Coulometric Sensor.

[29] Shankar S., Reddy J.P., Rhim J.W. (2015). Effect of lignin on water vapor barrier, mechanical, and structural properties of agar/lignin composite films. Int. J. Biol. Macromol..

[30] Pereira V.A., de Arruda I.N.Q., Stefani R. (2015). Active chitosan/PVA films with anthocyanins from Brassica oleraceae (Red Cabbage) as Time-Temperature Indicators for application in intelligent food packaging. Food Hydrocoll..

[31] El Mansouri N.E., Yuan Q., Huang F. (2011). Characterization of alkaline lignins for use in phenol-formaldehyde and epoxy resins. BioResources.

[32] Guo J., Ge L., Li X., Mu C., Li D. (2014). Periodate oxidation of xanthan gum and its crosslinking effects on gelatin-based edible films. Food Hydrocoll..

[33] Chaochanchaikul K., Jayaraman K., Rosarpitak V., Sombatsompop N. (2012). Influence of Lignin Content on Photodegradation in Wood/HDPE Composites Under UV Weathering. BioResources.

[34] Zhang W., Zhang Y., Cao J., Jiang W. (2021). Improving the performance of edible food packaging films by using nanocellulose as an additive. Int. J. Biol. Macromol..

[35] Lundgren E., Matos M., Avelino F., Lomonaco D., Rodrigues-souza I., Stephanie V., Gagosian C., Margarete M., Luiz W., Magalhães E. (2021). Safety aspects of kraft lignin fractions: Discussions on the in chemico antioxidant activity and the induction of oxidative stress on a cell-based in vitro model. Int. J. Biol. Macromol..

[36] Mujtaba M., Akyuz L., Koc B., Kaya M., Ilk S., Cansaran-Duman D., Martinez A.S., Cakmak Y.S., Labidi J., Boufi S. (2019). Novel, multifunctional mucilage composite films incorporated with cellulose nanofibers. Food Hydrocoll..

[37] Pereda M., Dufresne A., Aranguren M.I., Marcovich N.E. (2014). Polyelectrolyte films based on chitosan/olive oil and reinforced with cellulose nanocrystals. Carbohydr. Polym..

[38] Arrieta M.P., López de Dicastillo C., Garrido L., Roa K., Galotto M.J. (2018). Electrospun PVA fibers loaded with antioxidant fillers extracted from Durvillaea antarctica algae and their effect on plasticized PLA bionanocomposites. Eur. Polym. J..

[39] da Silva J.B.A., Nascimento T., Costa L.A.S., Pereira F.V., Machado B.A., Gomes G.V.P., Assis D.J., Druzian J.I. (2015). Effect of Source and Interaction with Nanocellulose Cassava Starch, Glycerol and the Properties of Films Bionanocomposites. Mater. Today Proc..

[40] Ferrer A., Pal L., Hubbe M. (2017). Nanocellulose in packaging: Advances in barrier layer technologies. Ind. Crops Prod..

[41] Struller C., Kelly P., Copeland N. (2019). Conversion of aluminium oxide coated films for food packaging applications—From a single layer material to a complete pouch. Food Packag. Shelf Life.

[42] Lange J., Wyser Y. (2003). Recent Innovations in Barrier Technologies for Plastic Packaging—A Review. Packag. Technol. Sci..

[43] Nair S.S., Yan N. (2015). Effect of high residual lignin on the thermal stability of nanofibrils and its enhanced mechanical performance in aqueous environments. Cellulose.

